# Case Report: Emergency awake craniotomy for cerebral abscess in a patient with unrepaired cyanotic congenital heart disease

**DOI:** 10.12688/f1000research.9722.2

**Published:** 2017-02-23

**Authors:** Corinne D’Antico, André Hofer, Jens Fassl, Daniel Tobler, Daniel Zumofen, Luzius A. Steiner, Nicolai Goettel

**Affiliations:** 1 Department of Anesthesia, Surgical Intensive Care, Prehospital Emergency Medicine and Pain Therapy, University Hospital Basel, University of Basel, Basel, Switzerland; 2 Department of Cardiology, University Hospital Basel, University of Basel, Basel, Switzerland; 3 Department of Neurosurgery, University Hospital Basel, University of Basel, Basel, Switzerland; 4 Department of Radiology, Division of Diagnostic and Interventional Neuroradiology, University Hospital Basel, University of Basel, Basel, Switzerland; 5 Department of Clinical Research, University Hospital Basel, University of Basel, Basel, Switzerland

**Keywords:** Awake craniotomy, congenital heart disease, conscious sedation

## Abstract

We report the case of a 39-year-old male with complex cyanotic congenital heart disease undergoing emergency craniotomy for a cerebral abscess. Maintenance of intraoperative hemodynamic stability and adequate tissue oxygenation during anesthesia may be challenging in patients with cyanotic congenital heart disease. In this case, we decided to perform the surgery as an awake craniotomy after interdisciplinary consensus. We discuss general aspects of anesthetic management during awake craniotomy and specific concerns in the perioperative care of patients with congenital heart disease.

## Introduction

Congenital heart disease (CHD) affects about 0.6% of newborns with a stable incidence over time ^1, 2^. Advances in surgical and medical treatment have shifted mortality largely to adulthood ^3^. Numbers of adult patients with CHD are steadily increasing, except in cohorts with Eisenmenger syndrome and unrepaired cyanotic defects ^4^. Therefore, surgeons and anesthesiologists are now facing more repaired survivors of CHD for noncardiac surgery ^5^. CHD patients are at high risk for long-term cardiac and noncardiac complications, and the perioperative management of these patients may be challenging ^6^. In this case report, we present the multidisciplinary management of a nighttime emergency awake craniotomy (AC) for stereotactic evacuation of an intracerebral abscess in an adult with unrepaired tricuspid atresia (TA) with palliative shunts.

## Case description

**Figure 1.**
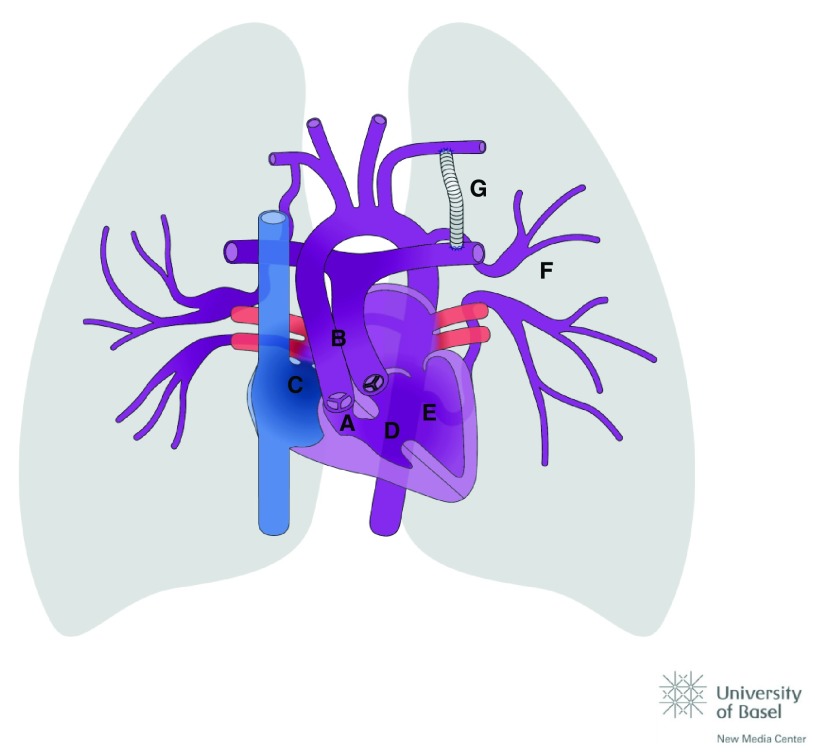
Schematic illustration of the patient’s cardiovascular anatomy showing ( **A**) tricuspid atresia, ( **B**) transposition of the great arteries, ( **C**) atrial septum defect, ( **D**) ventricular septum defect, ( **E**) single-ventricle physiology, ( **F**) major aorto-pulmonary collateral arteries, and ( **G**) Blalock-Taussig shunt. Copyright © 2014 New Media Center, University of Basel. All Rights Reserved.

A 39-year-old man (weight 75 kg; height 180 cm; body mass index 23 kg m ^-2^) presented to the emergency department at 7 *p.m.* with right frontal headache, fever, and paresthesia of the left side of the body. Nine days earlier, he underwent diode laser surgery for hypertrophic nasal turbinates under local anesthesia. The patient’s medical history revealed cyanotic CHD – a complex form of unrepaired TA. The patient received bilateral palliative Blalock-Taussig shunts in early childhood. The shunt on the left side was reported to be stenotic, and the right one was secondarily closed. A detailed illustration of the underlying cardiovascular anatomy is shown in Figure 1. In the past, the patient had suffered from bacterial endocarditis, pulmonary hemorrhage, renal and splenic infarctions, transitory ischemic attack and recurrent supraventricular tachycardia that were considered to be complications of his CHD. Regular oral medication consisted of metoprolol 50 mg, torasemide 10 mg and isotretinoin 10 mg once daily. An allergy to cephalosporins was noted.

Baseline peripheral oxygen saturation (SpO _2_) on room air was 80%. Examination of the patient’s hands revealed clubbed fingers with Hippocratic nails. Blood analysis showed secondary erythrocytosis (hemoglobin 210 g l ^-1^; hematocrit 0.62%) and mild leukocytosis (10.650 × 10 ^9^ l ^-1^). Serum C-reactive protein concentration was 47.8 mg l ^-1^. He was in sinus rhythm, and left ventricular function was mildly decreased with an ejection fraction of 46%.

**Figure 2.**
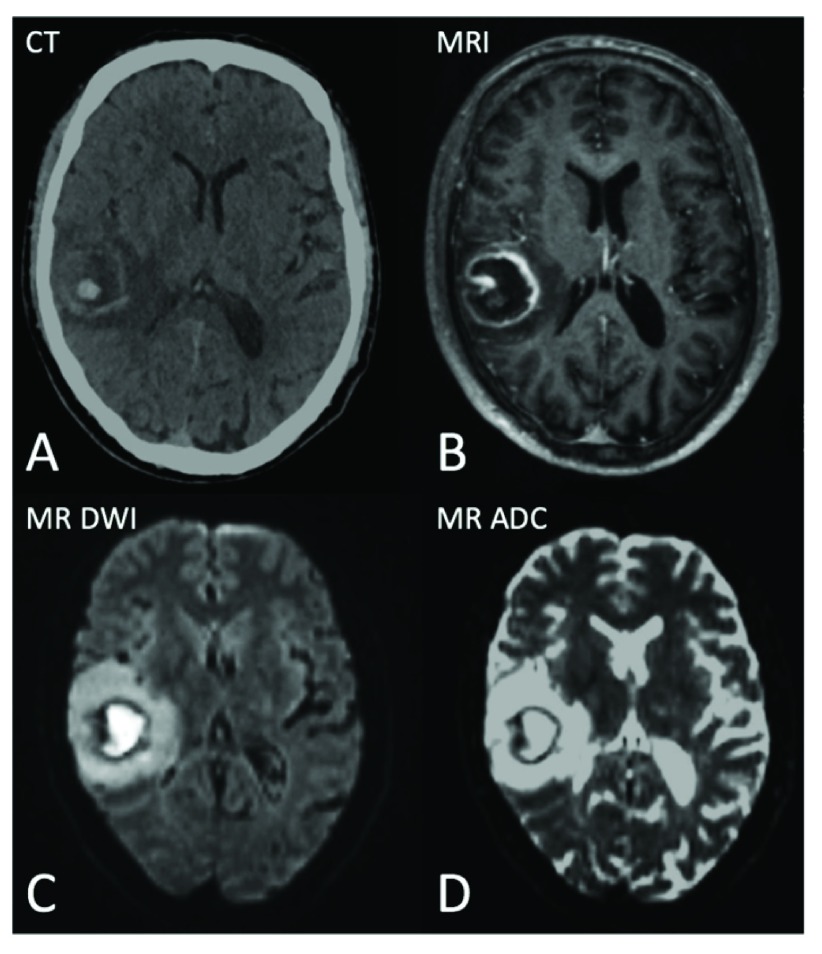
( **A**) Contrast-enhanced computed tomography (CT) scan and ( **B**) gadolinium-enhanced magnetic resonance imaging (MRI) revealed a 2.7 × 2.9 × 3.2 cm ring-enhancing lesion within the right superior temporal gyrus with significant surrounding edema and a small area of central hemorrhage. ( **C**) Diffusion-weighted imaging (DWI) and ( **D**) apparent diffusion coefficient (ADC) MRI showed a diffusion-restricted core, supporting the differential diagnosis of acute cerebral abscess.

Emergency contrast-enhanced computed tomography of the brain showed a ring-enhancing lesion within the right superior temporal gyrus. Subsequent Gadolinium-enhanced magnetic resonance imaging supported the differential diagnosis of an acute intracerebral abscess ( Figure 2). Based on these findings, emergency surgical evacuation of the abscess by computer-assisted stereotactic craniotomy was indicated. After interdisciplinary consensus involving the anesthetic and neurosurgical team, as well as the treating cardiologist, we decided to perform the procedure as an AC.

**Figure 3.**
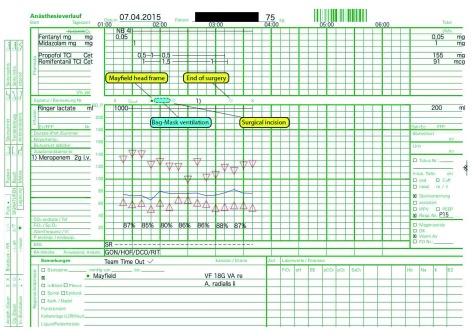
Intraoperative anesthesia record. For fixation of the patient’s head in the Mayfield frame, the target effect-site concentration (Cet) of propofol was temporarily increased to 1.0 µg ml ^-1^. Due to a subsequent brief loss of consciousness with upper airway obstruction, a short period of assisted bag-mask ventilation was performed. Adequate spontaneous respiration returned after the patient regained consciousness and was maintained throughout the procedure with supplemental oxygen delivery at 4 l min ^-1^ via nasal prongs (NB). Propofol and remifentanil infusions were adjusted to the patient’s clinical level of sedation and pain and bispectral index monitoring. During surgery, the patient remained in cardiac sinus rhythm (SR) and hemodynamically stable without any need for vasopressor support.

Upon arrival in the operating room, the patient was comfortably installed in the supine position with routine anesthesia monitoring (5-lead electrocardiogram, pulse-oximetry, noninvasive blood pressure monitoring). An arterial line was inserted in the left radial artery. The peripheral intravenous line was equipped with an air-eliminating filter to prevent paradoxical embolism. Supplemental oxygen at 4 l min ^-1^ was administered via nasal cannula to the spontaneously breathing patient. Expiratory carbon dioxide and respiratory rate were measured. Fentanyl 50 µg and midazolam 1 mg IV were administered during preparation for surgery. Prior to fixing the head in the Mayfield frame, conscious sedation was initiated using a target-controlled infusion (TCI, Injectomat TIVA Agilia, Fresenius Kabi AG, Oberdorf, Switzerland) of propofol and remifentanil with target effect-site concentrations (Cet) of 0.5 µg ml ^-1^ and 1.0 ng ml ^-1^, respectively. After increasing the Cet of propofol to 1.0 µg ml ^-1^ due to patient discomfort during head pinning, the patient lost consciousness for a short period of time. Bradypnoea and oxygen desaturation to a Sp0 _2_ of 80% occurred, and assisted mask-bag ventilation was required temporarily. The neurosurgeon then applied local anesthesia to the incision site using 20 ml of a 1:1 mixture of 0.5% bupivacaine and 1% lidocaine with 1:100,000 epinephrine. For remainder of the procedure, Cet of propofol (0.5 µg ml ^-1^) and remifentanil (0.5–1.5 ng ml ^-1^) were adjusted to the patient’s clinical level of sedation and pain and bispectral index monitoring. The patient was hemodynamically stable throughout the intervention. Respiratory rate stayed at 12–15 breath min ^-1^, and SpO _2_ ranged between 80 and 88%. Relevant intraoperative events are shown in Figure 3.

The patient’s left hemiparesthesia improved immediately following craniotomy and abscess decompression. Postoperatively, the patient was admitted to the intensive care unit. Some residual paresthesia was still present at discharge from the intensive care unit 10 hours later; however, neurological symptoms completely ceased by the second postoperative day. Bacteriological culture of the abscess fluid confirmed the diagnosis of a cerebral abscess and revealed *Streptococcus intermedius*. This was interpreted as hematogenous spread in the context of the previous turbinate surgery. The patient had received 2 g of meropenem IV intraoperatively. Under specific antibiotic treatment consisting of penicillin IV and oral metronidazole for 6 weeks, the abscess radiologically regressed. The patient was discharged home 14 days after the operation in stable condition.

## Discussion

It was important to understand the complex anatomy and underlying pathophysiology of the CHD for optimal anesthetic management of this patient. The predominant defect in this case is TA ( Figure 1). The tricuspid valve is absent, and the right ventricle is hypoplastic leading to a single-ventricle physiology. Transposed great arteries (TGA), as in this patient, are associated in about 30% of cases ^7^. In children with TA and TGA, pulmonary blood flow is usually elevated, unless the pulmonary valve is stenotic or artretic. Because no direct communication exists between the right atrium and the right ventricle, systemic venous return to the right atrium must be shunted to the left atrium through an atrial septal defect or patent *foramen ovale* (right-to-left shunt). Oxygen saturation values are equal in the aorta and the pulmonary artery due to complete mixing of systemic and pulmonary venous blood in the left ventricle. Pulmonary blood flow determines the degree of cyanosis and is influenced by the interplay of several factors such as the size of the ventricular septal defect, the presence or absence of pulmonary stenosis, as well as the patency of the ductus arteriosus. Most infants with TA require a palliative procedure ( *e.g.* Blalock-Taussig shunt) before definitive surgery can be performed ^8^. In this case, a Fontan palliation could not be performed, and survival was only possible due to decreased pulmonary blood flow.

Patients with CHD and chronic cyanosis may present a number of secondary pathophysiologic phenomena and are prone to cardiac and extracardiac complications, such as cardiac arrhythmias, thrombotic events, or bleeding disorders ^6, 9^. There is an increased risk for any kind of infection, including those of the central nervous system. Patients with new-onset headaches should be screened for cerebral abscess, which is a well-described complication of cyanotic CHD. The purpose of emergency surgery is to reduce the infectious burden, to decompress the adjacent brain, and to provide bacteriological samples that may guide antimicrobial therapy.

Patients with CHD, especially those with complex defects, have increased perioperative morbidity ^10, 11^. Additional risk factors for poor outcome in noncardiac surgery are emergencies and procedures involving the respiratory or central nervous system ^6, 9– 11^. The main objective in the management of this patient undergoing emergency craniotomy was to maintain pulmonary blood flow through the aorto-pulmonary anastomosis (Blalock-Taussig shunt) in order to provide optimal oxygen delivery, maintain systemic and pulmonary vascular resistance, and myocardial contractility ^9, 12^. We decided that these goals may best be achieved using a conscious sedation technique for AC. We considered that the myocardial depression and the drop in systemic vascular resistance associated with large doses of anesthetic agents during a general anesthetic could have compromised intraoperative hemodynamic stability in this high-risk patient.

AC has evolved into a standard of care for neurosurgical procedures that require awake functional mapping of the motor, sensory, visual, or language cortex when tumors are located in close proximity to eloquent areas of the brain, as well as for functional neurosurgery and epilepsy surgery. However, the practice of AC has spread to include routine procedures that do not involve awake functional cortical mapping or electrophysiological recording, *e.g.* stereotactic brain biopsy, ventriculostomy, or the evacuation of subdural hematomas. This is in part due to the implication of refined anesthetic management protocols and the use of effect-site controllable intravenous anesthetic agents such as propofol, dexmedetomidine and ultra-short acting opioids ( *e.g.* remifentanil). A recent systematic review showed that awake brain tumor resection led to a better perioperative neurological outcome compared with surgery under general anesthesia; moreover, AC was consistently associated with shorter hospital stay, less resource utilization, and high patient satisfaction ^13^. Major intraoperative complications during AC include respiratory depression, arterial hypertension, nausea and vomiting, air embolisms, brain swelling, seizures and loss of patient cooperation ^14^. Cautious patient selection focusing on airway assessment, ability to cooperate, risk of sedation failure and intraoperative surgical complications, as well as adequate preoperative psychological preparation of the patient are key elements for successful AC ^14^.

The evidence from the literature regarding the use of AC in cardiac patients is scarce. A recent case report describes the anesthetic management of an AC in a patient with cardiomyopathy and low cardiac output ^15^. The maintenance of intraoperative hemodynamic stability, indicated by a reduced use of vasopressors, seems to be facilitated during AC ^16^. In our patient, we favored AC primarily because of the underlying complex cyanotic CHD, and in order to preserve as much functional cardiovascular capacity as possible.

## Consent

Written informed consent for publication of the patients’ details and images was obtained from the patient.
