# Supplementary material for: Case Report: Emergency awake craniotomy for cerebral abscess in a patient with unrepaired cyanotic congenital heart disease
Source: F1000Res. 2017 Feb 23;5:2521. Originally published 2016 Oct 14. [Version 2] doi: 10.12688/f1000research.9722.2 (PMC5115221; doi:10.12688/f1000research.9722.2)
Supplement: Peer review history

# Peer review: Referee response for version 1

**DOI:** 10.5256/f1000research.10480.r17000

**Published:** 2016-11-16

**Authors:** Girija Prasad Rath

**Affiliations:** 1 Department of Neuroanaesthesiology and Critical Care, Neurosciences Centre, All India Institute of Medical Sciences (AIIMS), New Delhi, Delhi, India

It is an interesting case report of awake craniotomy with an indication to preserve functional cardiovascular capacity in an adult with congenital cyanotic heart disease. The case was well managed by the authors. The report may be indexed with following minor changes:

 Hemodynamic responses to various interventions or to painful responses to be described in graphical / picture format.

I have read this submission. I believe that I have an appropriate level of expertise to confirm that it is of an acceptable scientific standard.

# Peer review: Referee response for version 1

**DOI:** 10.5256/f1000research.10480.r17294

**Published:** 2016-11-01

**Authors:** Christopher Lysakowski

**Affiliations:** 1 Division of Anesthesiology, Geneva University Hospitals (HUG), Geneva, Switzerland

Concerning a case report of awake craniotomy in patient with cyanotic congenital heart disease (CHD).

 The authors describe an unusual case of a rather complicated and rare pathology that needs an emergency surgical intervention during a night. I have no specific scientific remarks concerning the content nor the design, but I think that for several reasons this case should be known by large anaesthesia community.

 First, the number of patients with CHD that can be scheduled for surgery is growing so our responsibility is to know how they should be handled, why spontaneous ventilation is preferable than mechanical, for which kind of complications we should be prepared etc.

 Second, we can always discuss which drugs should be used in a case of awake craniotomy, is dexmedetomidine better than other drugs? Personal experience and a local policy should be respected in such case.

 What is notable is that the authors were capable to establish, in this short time and during a night, an interdisciplinary consensus involving all disciplines, which was probably crucial to handle this case in this remarkable way.  

I have read this submission. I believe that I have an appropriate level of expertise to confirm that it is of an acceptable scientific standard.
